# IF1, a natural inhibitor of mitochondrial ATP synthase, is not essential for the normal growth and breeding of mice

**DOI:** 10.1042/BSR20130078

**Published:** 2013-09-17

**Authors:** Junji Nakamura, Makoto Fujikawa, Masasuke Yoshida

**Affiliations:** *Department of Molecular Bioscience, Kyoto Sangyo University, Kamigamo-Motoyama, Kyoto 603-8555, Japan; †International Cooperative Research Project (ICORP) ATP-Synthesis Regulation Project, Japan Science and Technology Agency (JST), 2-3-6 Aomi, Tokyo 135-0064, Japan; ‡Department of Biochemistry, Tokyo University of Science, 2641 Yamazaki, Noda 278-8510, Japan

**Keywords:** ATP synthesis, F_o_F_1_, knockout mouse, GAPDH, glyceraldehyde 3-phosphate dehydrogenase, KO, knockout, MEF, mouse embryonic fibroblast, ROS, reactive oxygen species, WT, wild-type

## Abstract

IF1 is an endogenous inhibitor protein of mitochondrial ATP synthase. It is evolutionarily conserved throughout all eukaryotes and it has been proposed to play crucial roles in prevention of the wasteful reverse reaction of ATP synthase, in the metabolic shift from oxidative phosphorylation to glycolysis, in the suppression of ROS (reactive oxygen species) generation, in mitochondria morphology and in haem biosynthesis in mitochondria, which leads to anaemia. Here, we report the phenotype of a mouse strain in which *IF1* gene was destroyed. Unexpectedly, individuals of this IF1-KO (knockout) mouse strain grew and bred without defect. The general behaviours, blood test results and responses to starvation of the IF1-KO mice were apparently normal. There were no abnormalities in the tissue anatomy or the autophagy. Mitochondria of the IF1-KO mice were normal in morphology, in the content of ATP synthase molecules and in ATP synthesis activity. Thus, IF1 is not an essential protein for mice despite its ubiquitous presence in eukaryotes.

## INTRODUCTION

Most of the ATP in aerobic cells is produced by ATP synthase in mitochondria coupled with proton flow driven by a proton motive force across membranes, which is generated by the respiratory chain. However, when the supply of oxygen or substrates for the respiratory chain becomes short due to ischaemia or other reasons, the ATP production becomes more dependent on glycolysis. At the same time, the back reaction of ATP synthase, futile ATP hydrolysis, should be prevented by any means possible, and IF1 has been proposed for this task. IF1 is a small protein that can bind to ATP synthase and its soluble subcomplex F_1_-ATPase, and inhibit their ATP hydrolysis activities [1]. It is a nuclear-coded mitochondrial protein and is evolutionarily conserved from yeast to mammals [2,3]. IF1 forms a dimer at acidic pH (~6.7) and exhibits inhibitory effects, but a tetramer form, which is formed at basic pH (~8.0), cannot interact with ATP synthase [4–6]. The crystal structure of the F_1_-ATPase with bound IF1 shows that the C-terminal α-helix of IF1 is inserted into the interface between the α- and β-subunits of F_1_-ATPase [7]. It is thought that when mitochondria lose proton motive force, the interior of the mitochondria become acidic and IF1 binds to ATP synthase to block wasteful ATP consumption [8]. Human cells with suppressed expression of IF1 have lower cellular ATP levels than control cells and produce higher levels of ROS (reactive oxygen species) [9,10]. When cells are deprived of both oxygen and glucose, IF1-knockdown cells die more rapidly than control cells [9]. Several groups have reported that IF1 facilitates the formation of the ATP synthase-dimer in the inner mitochondrial membranes and contributes to cristae formation [10–13], although this effect of IF1 on mitochondrial morphology remains controversial [9,14,15]. It has also been proposed that autophagy is facilitated in IF1-knockdown cells [16]. IF1 is abundantly expressed in human cancer cells, suggesting that it would play a critical role in the metabolic shift from oxidative phosphorylation to glycolysis [17,18]. A recent report showed that IF1 regulates haem synthesis in developing erythroblasts of zebra fish through the activity of ferrochelatase, which is sensitive to a pH alkaline shift in the mitochondria matrix space [19]. Furthermore, IF1 was found in human serum and was proposed to contribute to blood cholesterol metabolism [20,21]. Because of these wide-ranging and pronounced effects of IF1, mice without IF1-KO (IF1-knockout mice) are expected to have serious defects. However, we here show that the IF1-KO mouse is as healthy as the WT (wild-type) mouse. The actual role of IF1 in living mammals should be reconsidered.

## MATERIALS AND METHODS

### Generation of Atpif1-KO mice

The locus of the *IF1*-coding gene *Atpif1* of mice was disrupted by homologous recombination with a targeting vector in C57/BL6J ES cells, in which the neomycin cassette replaced the first, second and a part of the third exons encompassing the full-length coding sequence of *Atpif1*. The targeting vector was constructed by incorporating the 5′ NotI-XhoI 2.5-kb fragment and the 3′ SmaI-SalI 6.0-kb fragment amplified from Bac clones by PCR into a vector that contained the neomycin-resistant gene and a diphtheria toxin A subunit in tandem (Supplementary Figure S1A; available at http://www.bioscirep.org/bsr/033/bsr033e067add.htm). This construct was linearized with NotI and electroporated into the C57BL/6J-derived ES cells. Targeted ES cells screened by Southern blotting and PCR analysis were injected into blastocysts from Balb/c mice for the generation of chimaeric mice. Chimaeric male mice were mated with C57BL/6J female mice and the agouti-coloured offspring were analysed for germline transmission. Mice were genotyped by Southern blotting and PCR analysis. Genomic DNA was digested with ApaLI and then hybridized with a 5′ external probe flanking the 5′ region of the targeting vector. The WT allele gives a band of 7.6 kb, whereas the correctly targeted mutant allele shows a band of 15.8 kb (Supplementary Figure S1B). PCR analysis was performed using the following primer set: the 5′ (GTGCATGTGCACTTTTGTGTGTGTGTATGC) and 3′ (ACACAGCAGCTCACAAGCACATGTAACTGC) primers located in the second intron (300 bp for the WT *Atpif1* allele), and the 5′ (GGCTATGACTGGGCACAACAGACAATCGGC) and 3′ (CATGATATTCGGCAAGCAGGCATCGCCATG) primers located in the neomycin resistance gene (531 bp for the mutated allele) (Supplementary Figure S1C). Mice that carried one copy of the deleted gene were interbred to generate litters and WT, heterozygous and homozygous genotypes were determined by Southern blotting and PCR analysis of DNA extracted from the tails. *In situ* hybridization analysis of embryos at embryonic day 16.5 (E16.5) of each genotype showed that *Atpif1* mRNA was reduced by <50% in heterozygous embryos compared with WT embryos and could not be detected in *Atpif1*-KO (IF1-KO) embryos (Supplementary Figure S1D). To confirm the absence of the IF1 protein in IF1-KO mice, immuno-fluorescence analysis of WT and IF1-KO MEFs (mouse embryonic fibroblasts) was performed. In WT MEFs, IF1 was localized in the mitochondria. In contrast, IF1 was undetectable in IF1-KO MEFs (Supplementary Figure S1E). All animal experiments were carried out according to the correct institutional procedures.

### Mitochondrial ATP synthesis and hydrolysis activity

Mitochondria were prepared by differential centrifugation from the mouse liver, as described previously [22]. The tissues were homogenized in ice-cold buffer containing 250 mM sucrose, 0.1 mM EGTA and 20 mM Tris/HCl, pH 7.4. The homogenate was centrifuged at 1000 ***g*** for 10 min at 4°C and the supernatant was centrifuged at 10000 ***g*** for 10 min at 4°C. The precipitate was washed and resuspended in a minimal volume of buffer containing 250 mM sucrose, 0.1 mM EGTA and 20 mM Tris/HCl, pH 7.4. The suspension was subjected to sonication (200 W, 30 s, 10 times) and centrifuged at 10000 ***g*** for 10 min at 4°C. The supernatant fraction was used for assays. The protein concentration was determined by the Bradford assay.

The mitochondrial ATP synthesis activity was measured at 25°C by monitoring the increase in absorbance at 340 nm of NADPH in the presence of hexokinase and gulucose-6-phosphate dehydrogenase [23]. The reaction was started by addition of the mitochondria suspension to a reaction mixture containing 10 mM K_2_HPO_4_, 2 mM MgCl_2_, 10 mM succinate, 1 mM glucose, 50 mM Tris/HCl, pH 7.4, 1 mM ADP, 0.2 mM NADP^+^, 4.1 international unit hexokinase/2.0 international unit glucose-6-phosphate dehydrogenase (Roche). ATP hydrolysis activity was measured at 25°C using a coupled assay to follow the oxidation of NADH at 340 nm by pyruvate kinase and lactic dehydrogenase reactions [24]. The reaction was started by addition of the mitochondria suspension to a reaction mixture containing 100 mM KCl, 1 mM MgCl_2_, 1 mM phosphoenolpyruvate, 50 mM Tris/HCl, pH 7.4, 1 mM ATP, 0.2 mM NADH and 3.6–6.0 international unit pyruvate kinase/5.4–8.4 international unit LDH (lactate dehydrogenase) (Sigma).

### Cell culture

Embryonic day 14.5 (E14.5) embryos from WT and IF1-KO mice were used to generate MEFs. Briefly, the head, limbs and viscera were removed from the embryos, and the carcasses were minced and then trypsinized in PBS containing 0.25% (w/v) trypsin and 1 mM EDTA for 10 min at 37°C. The cells were collected and grown at 37°C in 5% (v/v) CO_2_ in DMEM (Dulbecco's modified Eagle medium; Sigma) supplemented with 10% (v/v) FBS (fetal bovine serum; Gibco BRL) and penicillin–streptomycin.

### Immunoblotting

Antibodies against the α (sc-53613)-, β (A21351)-subunit of ATP synthase and LC3 (PM036) were purchased from Santa Cruz Molecular Probes. The antibody against LC3 (PM036) was purchased from Medical and Biological Laboratories. Secondary antibodies labelled with alkaline phosphatase were from Jackson ImmunoResearch Laboratories. Proteins in the tissues and MEFs were treated with 1% (v/v) NP40 and the solubilized extracts (20 μg protein) were applied to PAGE in the presence of 0.1% (w/v) SDS. Immunoblotting was carried out by standard procedures. Blots were visualized with 5-bromo-4-chloro-3-indolylphosphate and NBT (Nitro Blue Tetrazolium)-buffered substrate tablets (Sigma). The quantification was performed using MultiGauge software (Fujifilm), and the signal was normalized to the amount of GAPDH (glyceraldehyde 3-phosphate dehydrogenase; HyTest, 5G4).

### Clear native PAGE

Mitochondria from the livers of mice were suspended in solubilization buffer containing 50 mM Bis/Tris, pH 7.2, 0.1 mM EDTA, 50 mM NaCl, protease inhibitor cocktail (Nacalai) and digitonin, and incubated for 10 min on ice. For solubilization of mitochondrial membrane proteins, the optimal ratio between protein and digitonin of 6 g/g was used. The insoluble materials were removed by centrifugation at 100000 ***g*** for 10 min at 4°C. The collected supernatant was supplemented with 5% (v/v) glycerol and 0.01% Ponceau S and 5 μg of proteins was loaded onto a 3–12% gradient native gel (Invitrogen) for the high-resolution Clear Native PAGE [25]. The transfer of native gels was carried out as described above.

### Microscopy

For optical microscopy, the cells were fixed with 4% (v/v) PFA (paraformaldehyde) and permeabilized with 50 μg/ml digitonin. The blocking reagent was 2% (v/v) normal goat serum. After incubation with primary antibodies, the cells were incubated with Alexa Fluor 488-labelled goat anti-rabbit IgG (Molecular Probes). Observation was carried out with a Biozero BZ-8000 (Keyence). For electron microscopy, liver tissue samples from 3-month-old WT and IF1-KO mice were fixed with 2% (w/v) glutaraldehyde in 0.1 M PBS, postfixed with 2% (w/v) osmium tetroxide followed by dehydration through a graded series of ethanol, and embedded in Quetol-812 resin. Ultrathin sections were cut on a Reichert Ultracut E, stained with 2% (w/v) uranyl acetate followed by lead citrate, and viewed on a JEM1200EX (Jeol) transmission electron microscope at 80 kV.

### Statistical analysis

The data are expressed as the mean±S.E.M. Comparisons between two groups were analysed using the Student *t* test. Differences of *P*<0.05 (* in the graph) were considered to be statistically significant, and those of *P*>0.05 non-significant (N.S. in the graph).

## RESULTS AND DISCUSSION

### Growth, breeding and behaviours of the IF1-KO mice

**Figure 1:**
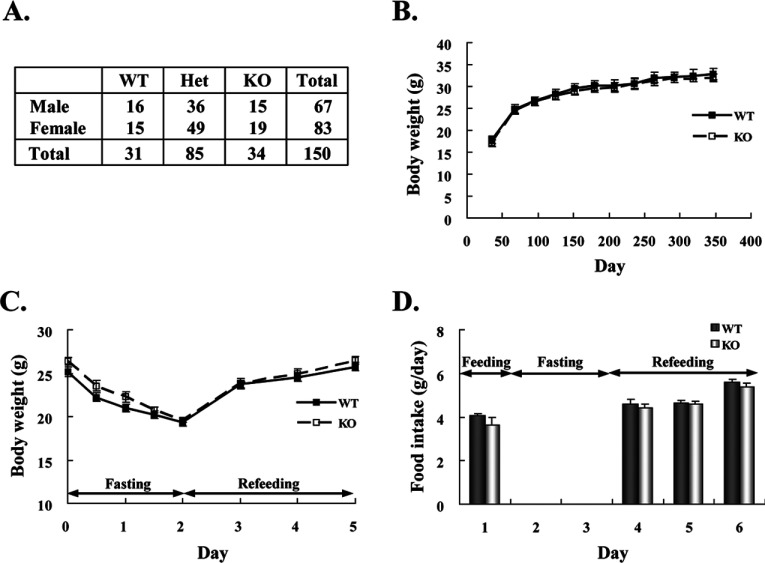
Breeding, growth and starvation of IF1-KO mice (**A**) Genotype of progeny from *Atpif1* heterozygous (Het) intercross. Genomic DNA of 3-week-old offspring was extracted from the tails and analysed by PCR. (**B**) Growth curves of WT and IF1-KO mice. The weights of male (WT [*n*=7], IF-KO [*n*=7]) littermates were measured weekly after weaning. (**C**) Body weight change of 3-month-old mice (WT [*n*=7], IF1-KO [*n*=5]) during and after 48-h fasting. (**D**). Food intake before, during and after 48-h fasting.

When heterozygous mutants were intercrossed, 150 viable offspring were obtained (Figure 1A). Mice of each genotype were born in an approximately Mendelian ratio, that is, WT (21%), heterozygotes (56%) and IF1-KO (23%), suggesting that the loss of IF1 does not affect the breeding or development of mice. To assess the effects of the absence of IF1 on growth, WT and IF1-KO male mice were weighed through the ages of 35 and 350 days (Figure 1B). Within this time period, the growth curves for WT and IF1-KO male mice overlapped. Similar results were obtained for female mice (not shown). The IF1-KO mice grew normally with no behavioural abnormalities for at least 1 year. As IF1 is able to prevent the reverse reaction of ATP synthase and is expected to save ATP under starvation conditions, the response to fasting of WT and IF1-KO mice was examined (Figure 1C). Mice underwent 48-h fasting and then were fed again. The loss of body weight during the fasting period and recovery of body weight after free refeeding observed for IF1-KO mice appeared to be the same as those observed for WT mice. The amount of food intake by IF1-KO mice after the fasting period was also the same as that by WT mice. It was reported that IF1 regulates haem synthesis in developing erythroblasts of zebra fish [19]. However, IF1-KO mice show normal blood profiles and signs of anaemia are not found (Supplementary Table S1; available at http://www.bioscirep.org/bsr/033/bsr033e067add.htm).

### Tissues, cells and mitochondria of the IF1-KO mice

**Figure 2:**
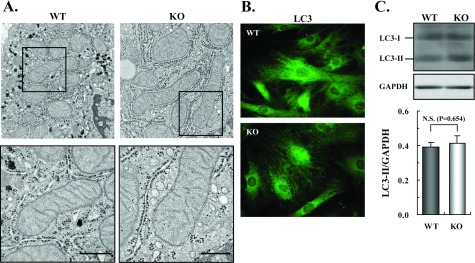
Mitochondrial morphology and autophagy in IF1-KO mice (**A**) Electron micrographs of mitochondria in liver cells from 3-month-old WT and IF1-KO mice. Lower panels are higher magnification. Scale bars, 0.5 μm. (**B**) Images of WT and IF1-KO MEFs stained with antibody against an autophagy-specific factor LC3. (**C**) Analysis of LC3-I and LC3-II in WT and IF1-KO MEFs. Upper panel. SDS/PAGE stained by anti-LC3 antibody. Whole cell extract was applied to SDS/PAGE and immunoblotted with anti-LC3 antibody. A band of GAPDH in the same gel stained with its antibody is shown as a control of the applied protein amount. Lower panel. Band intensity of LC3-II relative to that of GAPDH (WT [*n*=6], IF1-KO [*n*=6]).

Histological examination of the thymus, lung, heart, stomach, pancreas, liver, kidney and testis of IF1-KO mice did not show apparent changes (Supplementary Figure S2; available at http://www.bioscirep.org/bsr/033/bsr033e067add.htm). We observed organelles of liver cells from IF1-KO mice with an electron microscope, but no obvious changes from the corresponding WT mice were observed (Figure 2A, upper panel). Unlike in the previous reports [10–12], the morphology of mitochondria of IF1-KO mice, including the development of cristae structure, appeared to be the same as that of WT mice (Figure 2A, lower panel). Next, we examined whether autophagy was facilitated in the absence of IF1 [16]. When IF1-KO MEFs were stained with antibody against LC3, a specific protein factor required for the generation of autophagosomes [26], the images of IF1-KO MEFs were very similar to those of WT MEFs (Figure 2A). In the generation of autophagosomes, LC3 was processed to LC-I by proteolytic cleavage and then to LC-II by the attachment of phosphatidylethanol amine. Total proteins from WT and IF1-KO MEFs were analysed with SDS/PAGE stained with anti-LC3 antibody. The amounts of LC3-I and LC3-II in IF1-KO MEFs, as compared with the internal standard of glyceroaldehyde 3-phosphate, were very similar to those of the WT MEFs (Figure 2C). We conclude that autophagy was not significantly stimulated in IF1-KO MEFs.

### ATP synthase of the IF1-KO mice

**Figure 3:**
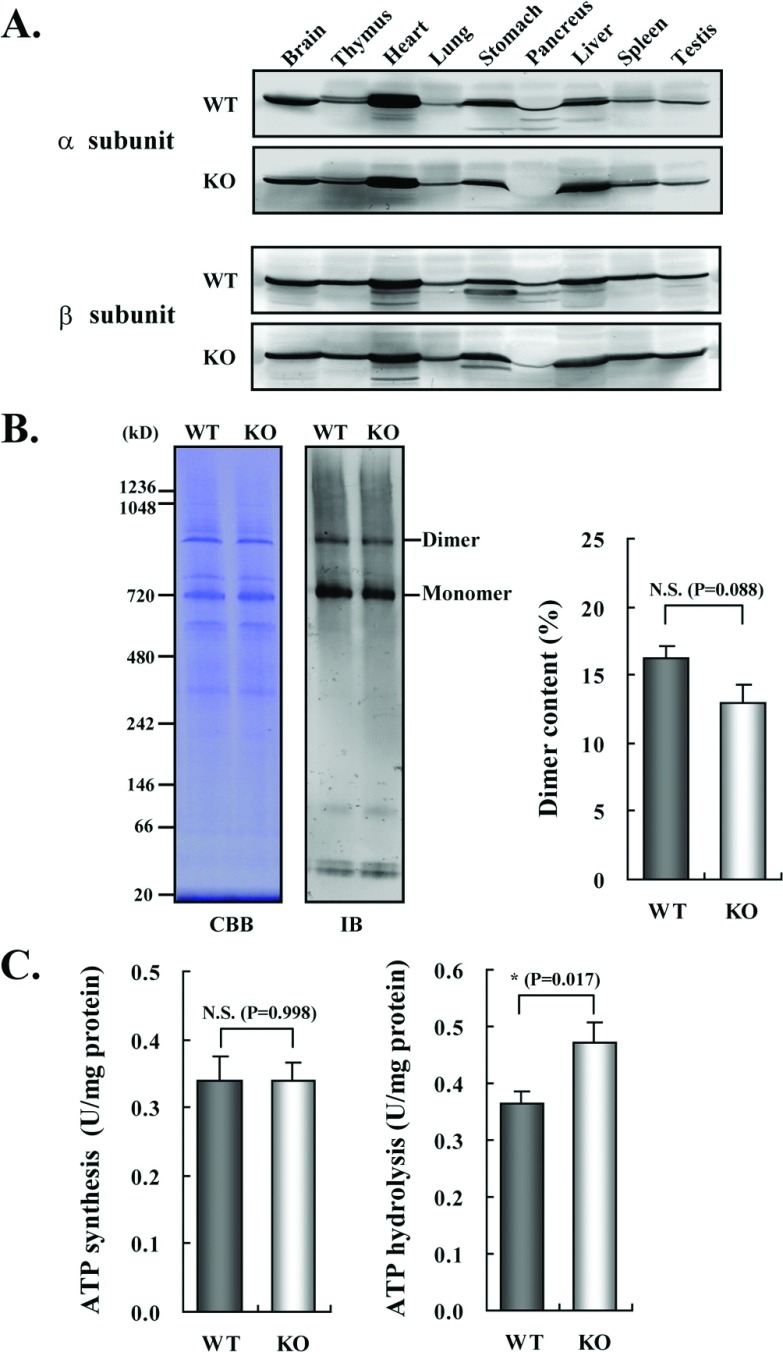
Expression and activities of ATP synthase in IF1-KO mouse (**A**) Expression of α- and β-subunits of ATP synthase in various tissues. Whole protein extracts of different tissues from 3-month-old WT and IF1-KO mice were analysed by SDS/PAGE followed by immunoblotting. (**B**) Content of the monomer and dimer forms of ATP synthases in mitochondria. Left panel. Clear Native PAGE. The solubilized mitochondrial protein (5 μg) of the hearts from 8-month-old WT and IF1-KO mice were subjected to Clear Native PAGE and immunoblotted with anti-β subunit of ATP synthase antibody. Another gel was stained with CBB (Coomassie Brilliant Blue) to show that equal amount of proteins were loaded to PAGE. The positions of molecular mass markers are indicated on the left. Right panel. WT (*n*=6) and IF1-KO (*n*=5) mice were subjected to the analysis and band intensities were quantified. Dimer content (%) is expressed as 100×dimer/(monomer+dimer). (**C**) Mitochondrial ATP synthesis and hydrolysis activities. Mitochondria were prepared from livers of 4-month-old WT (*n*=6) and IF1-KO (*n*=5) mice.

Next, we examined whether IF1 deficiency affects the expression of ATP synthase. As shown in Figure 3A, the expression levels of the α- and β-subunits of ATP synthase in various organs of IF1-KO mice were not changed from those of WT mice. The amount of ATP synthase molecules, assessed by Clear Native PAGE, was also unchanged. Clear Native PAGE further revealed that the content ratio of the dimer form of ATP synthase molecules in the heart from IF1-KO mice was not significantly different from that observed for WT mice (Figure 3B). Thus, facilitation of the dimer formation that results in the cristae formation by IF1 [10–13] was not confirmed in living animals. The ATP synthesis and hydrolysis activities of the liver mitochondria of IF1-KO mice were compared with those of WT mice (Figure 3C). The results showed that the level of ATP synthesis driven by succinate oxidation was almost the same between IF1-KO and WT mice. A level of mitochondrial ATP hydrolysis activity of IF1-KO mice was slightly higher than that of the WT mice but the difference was not large.

### Concluding remarks

As the importance of IF1 has been suggested from the results of several *in vitro* and *in vivo* experiments, we anticipated that serious defects would be observed in IF1-KO mice in the present study. However, as reported here, the mice lacking IF1 showed no defective phenotype under the present conditions. This result was surprising but not completely unexpected, since IF1 is also non-essential for *Caenorhabditis elegans* [our unpublished results, 27] and for yeast [28]. In addition, IF1-knockdown HeLa cells and control cells apparently show the same cell growth, glucose consumption, mitochondrial ATP synthesis and mitochondria morphology [9]. One of the possible explanations for the non-essentiality of IF1 is that other as-yet-unknown proteins may take over the functions of IF1 in IF1-KO mice. This contention, however, is rather unlikely, if not impossible, because one of the two genes for the same function, in general, would have easily been lost during evolution, while IF1 is well conserved from yeast to humans. A more likely possibility is that IF1 becomes essential when the animal encounters stressful situations that we have not tested in the laboratory. That is, while cells can manage without IF1 in daily life, they may depend on it under certain extreme conditions.

## Online data

Supplementary data
