# Supplementary material for: IF1, a natural inhibitor of mitochondrial ATP synthase, is not essential for the normal growth and breeding of mice
Source: Biosci Rep. 2013 Sep 17;33(5):e00067. doi: 10.1042/BSR20130078 (PMC3775512; doi:10.1042/BSR20130078)
Supplement: Supplementary data [file bsr033e067add.pdf]

Biosci. Rep. (2013) / **33** / art:e00067 / doi 10.1042/BSR20130078

OPEN ACCESS

Original Paper

SUPPLEMENTARY DATA

IF1, a natural inhibitor of mitochondrial ATP synthase, is not essential for the normal growth and breeding of mice

Junji NAKAMURA*†, Makoto FUJIKAWA†‡ and Masasuke YOSHIDA*†[1]

*Department of Molecular Bioscience, Kyoto Sangyo University, Kamigamo-Motoyama, Kyoto 603-8555, Japan, †International Cooperative Research Project (ICORP) ATP-Synthesis Regulation Project, Japan Science and Technology Agency (JST), 2-3-6 Aomi, Tokyo 135-0064, Japan, and ‡Department of Biochemistry, Tokyo University of Science, 2641 Yamazaki, Noda 278-8510, Japan

Table S1 Haematological parameters of WT and IF1-KO mice
Peripheral blood samples were obtained from 1-year-old WT ($n = 7$) and IF1-KO ($n = 6$) mice. There were no significant differences in haematological parameters between the WT and IF1-KO mice. All data are expressed as the mean±S.E.M.

Parameters	WT	KO	Differences
WBC ($\times 10^2/\mu$l)	82.4±8.5	76.7±7.7	N.S. ($P = 0.628$)
RBC ($\times 10^5/\mu$l)	92.7±1.4	90.9±1.7	N.S. ($P = 0.418$)
HGB (g/dl)	13.5±0.2	13.1±0.3	N.S. ($P = 0.137$)
HCT (%)	49.6±0.7	47.9±1.3	N.S. ($P = 0.301$)
MCV (fl)	53.5±0.3	52.7±0.7	N.S. ($P = 0.353$)
MCH (pg)	14.6±0.1	14.4±0.2	N.S. ($P = 0.315$)
MCHC (%)	27.3±0.1	27.3±0.2	N.S. ($P = 0.905$)
PLT ($\times 10^4/\mu$l)	92.8±9.0	80.7±5.3	N.S. ($P = 0.275$)

The haematological parameters are abbreviated as: WBC, white blood cell; RBC, red blood cell; HGB, haemoglobin; HCT, haematocrit; MCV, mean corpuscular volume; MCH, mean corpuscular haemoglobin; MCHC, mean corpuscular haemoglobin concentration; PLT, platelets.

[1] To whom correspondence should be addressed (email masasuke.yoshida@cc.kyoto-su.ac.jp).

© 2013 The Author(s) This is an Open Access article distributed under the terms of the Creative Commons Attribution Licence (CC-BY) (http://creativecommons.org/licenses/by/3.0/) which permits unrestricted use, distribution and reproduction in any medium, provided the original work is properly cited.

Bioscience Reports www.bioscirep.org

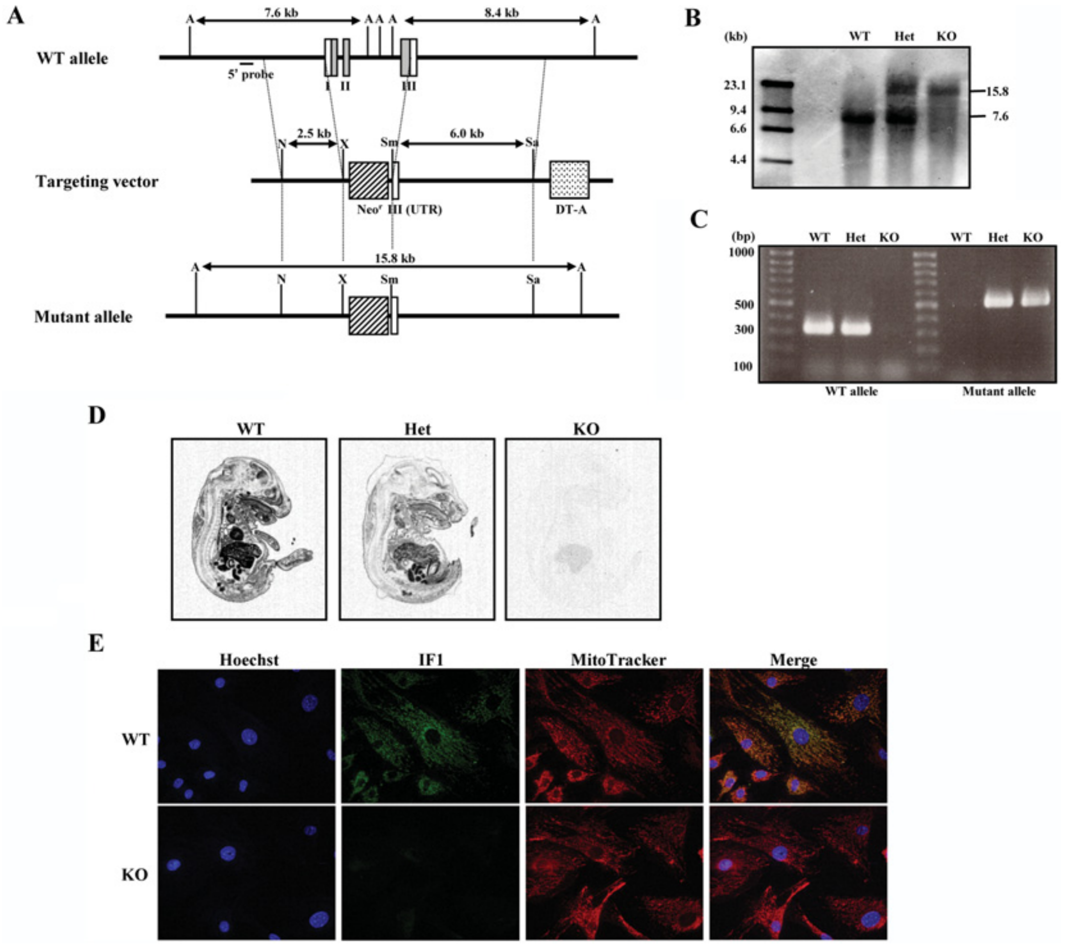

Figure S1 **Generation of IF1-KO mice**

(**A**) The endogeneous mouse *Atpif1* locus, the targeting vector and the mutant *Atpif1* locus are shown schematically. The three exons of *Atpif1* are represented by white boxes, and grey shading indicates the coding regions. The targeting vector consists of the 5′ homology region, Neo^r (neomycin phosphoribosyltransferase), 3′ homology region and, at the 3′ end, DT-A (diphtheria toxin A) for negative selection. Restriction enzymes are abbreviated as follows: A, ApaLI; N, NotI; Sa, SalI; Sm, SmaI; X, XhoI. (**B**) Genotype analysis by Southern blotting. Genomic DNA obtained from the tails of WT (wild-type), Het (heterozygous) and homozygous (KO) mice littermates was digested with ApaLI and hybridized with the 5′ external probe shown in A. The 7.6 kb band derived from the WT allele and the 15.8 kb band specific for the mutant allele are indicated on the right. (**C**) Genotype analysis by PCR. The 300 and 531 bp bands represent the products of primers from the WT and mutant alleles, respectively. (**D**) *In situ* hybridization analysis of the *Atpif1* gene in *Atpif1* mutant embryos. The coronal cryosections of embryos at E16.5 of each genotype were analysed for *Atpif1* mRNA expression by *in situ* hybridization. A 399 bp mouse *Atpif1* cDNA probe was subcloned into pBluescript and linearized by EcoRI to generate the antisense probe. Hybridization with the [α-^{35}S] UTP (PerkinElmer)-labelled probes was performed at 55 °C for 16 h. The sections were treated with RNaseA for 30 min, and then washed twice sequentially in 2×SSC for 10 min and 0.2×SSC for 30 min at 55 °C, respectively. The radioactivity of each section was measured using a phosphor image analyser (GE Healthcare). *Atpif1* mRNA was not detected in the sections of IF1-KO embryos. (**E**) Immunofluorescence analysis of WT and IF1-KO mouse embryonic fibroblasts (MEFs) using anti-IF1 antibody (green), (Molecular Probes, A21355). Mitochondria and nuclei were counter-stained with MitoTracker Red CMXRos (red), (Molecular Probes) and Hoechst33342 (blue), respectively.

© 2013 The Author(s) This is an Open Access article distributed under the terms of the Creative Commons Attribution Licence (CC-BY) (http://creativecommons.org/licenses/by/3.0/) which permits unrestricted use, distribution and reproduction in any medium, provided the original work is properly cited.

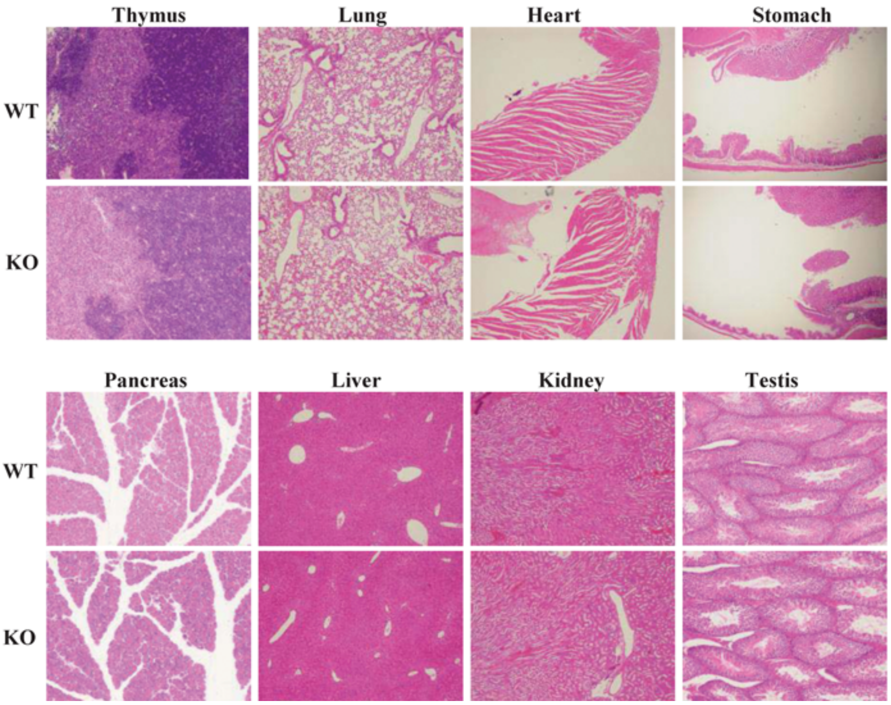

Figure S2 Histological analysis of different tissues from WT and IF1-KO mice
Different tissues from 3-month-old WT and IF1-KO mice were fixed in 10% (v/v) formalin. The fixed samples were then washed, dehydrated, cleared and embedded in paraffin. The embedded samples were sectioned at 5-μm thickness. They were mounted onto glass slides, deparaffinized, and stained with haematoxylin–eosin. No histological and morphological abnormalities in the IF1-KO mice were detected compared with their WT littermates.

Received 12 July 2013; accepted 24 July 2013

Published as Immediate Publication 28 July 2013, doi 10.1042/BSR20130078

© 2013 The Author(s) This is an Open Access article distributed under the terms of the Creative Commons Attribution Licence (CC-BY) (http://creativecommons.org/licenses/by/3.0/) which permits unrestricted use, distribution and reproduction in any medium, provided the original work is properly cited.
